# Esophageal carcinosarcoma comprising undifferentiated pleomorphic sarcoma and squamous cell carcinoma: a case report

**DOI:** 10.1186/s13019-022-01957-w

**Published:** 2022-08-26

**Authors:** Ziyao Fang, Tian Xia, Shu Pan, Chun Xu, Sheng Ju, Ziqing Shen, Jun Zhao

**Affiliations:** Department of Thoracic Surgery, The First Affiliated Hospital of Soochow University, Pinghai Road, Suzhou, 215000 Jiangsu China

**Keywords:** Esophagus, Carcinosarcoma, Case report, Video assisted thoracic surgery (VATS)

## Abstract

**Background:**

Esophageal carcinosarcoma (ECS) is a rare malignant tumor that often presents as an intraluminal polypoid lesion in the esophageal lumen. The pathogenesis of esophageal carcinosarcoma is not clear and its etiology is still being discussed.

**Case presentation:**

We report the case of a 68-year-old male who had dysphagia for approximately three months. Contrast-enhanced computed tomography showed an irregular enhancing mass in the lower esophagus. Endoscopy showed a gray-white mass with a smooth surface that almost filled the esophageal lumen at a location 28 cm from the incisor tooth. Considering the location of the tumor, we opted for Ivor-Lewis esophagectomy with intrathoracic anastomosis through a 5-port laparoscope and uniport video-assisted thoracic surgery (VATS). Pathological analysis showed that the mass comprised carcinoma in situ and pleomorphic sarcoma, without lymphatic metastasis. The postoperative pathological stage was T1bN0M0, stage I (Japanese Classification of Esophageal Cancer 11th Edition). The latest follow-up of the patient was 14 months after the surgery, and no signs of recurrence or metastasis were found.

**Conclusion:**

This case demonstrates a rare esophageal malignancy with a peculiar histological composition. Successful VATS esophagectomy with intrathoracic anastomosis was conducted without recurrence or metastasis at the 14-month follow-up.

## Introduction

Esophageal carcinosarcoma (ECS) is regarded as a rare malignant tumor that is biphasic in nature, and is composed of both carcinomatous and sarcomatous elements [1]. Various diagnostic nomenclatures, such as spindle cell squamous cell carcinoma, sarcomatoid carcinoma and squamous cell carcinoma with sarcomatoid changes, have been assigned to this particular tumor [1]. ECS often presents as a bulky, large intraluminal polypoid lesion with distinct pathological features; the carcinomatous elements are located at the base of the polypoid tumor, while the sarcomatoid elements are located at the body of the polypoid tumor [2]. Compared with esophageal squamous cell carcinoma (ESCC), ECS seems to have a better prognosis because of its limited invasiveness. However, the recurrence rate of ECS is still high with a five-year survival rate similar to that of esophageal squamous cell carcinoma [3, 4]. Complete tumor resection with regional lymphadenectomy is recommended for the surgical management of esophageal sarcoma [5].

## Case report

A 68-year-old male patient presented to the thoracic surgery department complaining of dysphagia for approximately three months. The patient had begun to experience choking sensations when swallowing solid items three months previously. He could only consume semisolid items when he presented to our hospital and sometimes experienced vomiting or reflux after eating. His history of past illnesses included hypertension for more than ten years and diabetes for more than six years. The patient had been taking nifedipine (20 mg bid) to control his blood pressure and glipizide (5 mg tid) to control his plasma glucose level. He denied odynophagia, retrosternal chest pain and weight loss. No specific family history was mentioned by the patient.

**Fig. 1:**
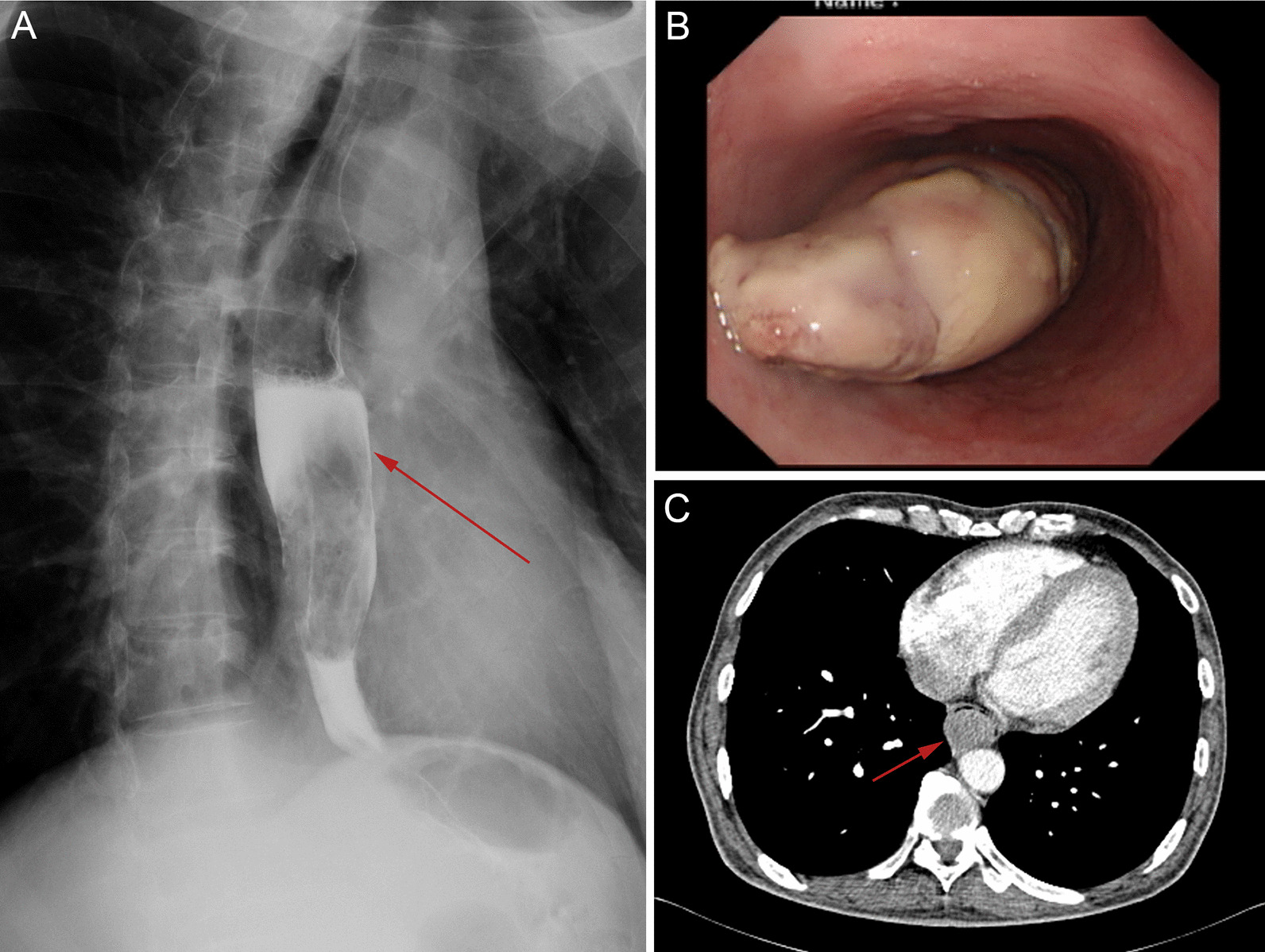
Radiographic and endoscopic examination of the mass. **A** X-ray examination shows an irregular approximately circular filling defect. **B** Endoscopic examination showed a gray-white mass in the esophageal lumen that was connected to the esophageal wall via a pedicle. **C** Contrast-enhanced computed tomography revealed a large mass obstructing the esophageal lumen without enlarged lymph nodes or invasion to the adjacent organs

An X-ray barium meal test revealed an irregular, approximately circular filling defect, with a diameter of 8 cm, in the mid-inferior part of the esophagus, accompanied by stenosis and stiffness of the esophageal cavity (Fig. 1A). Endoscopy indicated that the mass was gray-white with a smooth surface, was attached to the posterior esophageal wall via a pedicle, and was located 28 cm from the incisor tooth. The mass almost filled the esophageal lumen, and the endoscope body was not able to pass through the mass (Fig. 1B). Contrast-enhanced computed tomography (CT) revealed an irregular enhancing soft-tissue density mass without evidence of invasion into the adjacent structures. No apparent lymphatic metastasis or distant metastasis was reported (Fig. 1C). Histopathological examination of an endoscopic biopsy specimen indicated inflammatory necrotic tissue in the esophageal mucosa.

Based on the location of the tumor, the patient was treated with Ivor-Lewis esophagectomy with intrathoracic anastomosis. The abdominal stage was performed with a 5-port technique, and the thoracic stage was performed with the uniport video-assisted thoracic surgery (VATS). The peritoneum covering the pancreas was dissected above the common hepatic artery, and the upper abdominal lymph nodes were exposed and dissected. The left gastric vessels were exposed and transected. The lymph nodes along the left gastric artery were also dissected. The gastrocolic ligament was transected along the greater curvature of the stomach. The left gastroepiploic and short gastric vessels were exposed and transected. The gastric fundus was sequentially mobilized. Then a 5-cm wide gastric tube was created with a linear endoscopic stapler. The right crus of the diaphragm was transected to allow access to the posterior mediastinum. The retropericardial segment of the esophagus was then mobilized with the surrounding tissue and lymph nodes. For the thoracic stage, the patient was placed in the left lateral decubitus position, and an incision was made at the sixth intercostal space on the right side. The esophagus was mobilized by dissection of the soft tissue, including the lung, pericardium, aorta and pleura. Lymphadenectomy of the mediastinum was carried out at the same time. The esophagus was resected with scissors 3 cm above the tumor. Then, the gastric conduit was pulled into the pleural cavity through the hiatus, and an incision was made in the proximal end of the stomach tube to place the handle of the end-to-end anastomosis stapler. The anvil was delivered to the incisal edge of the esophagus as the anastomosis point through a nasogastric tube by the anesthesiologist. Finally, the stapler was connected to the anvil, and the anastomosis was completed. The anastomosis was covered by fatty tissue, and drainage tubes were placed into the pleural cavity and the mediastinum.

**Fig. 2:**
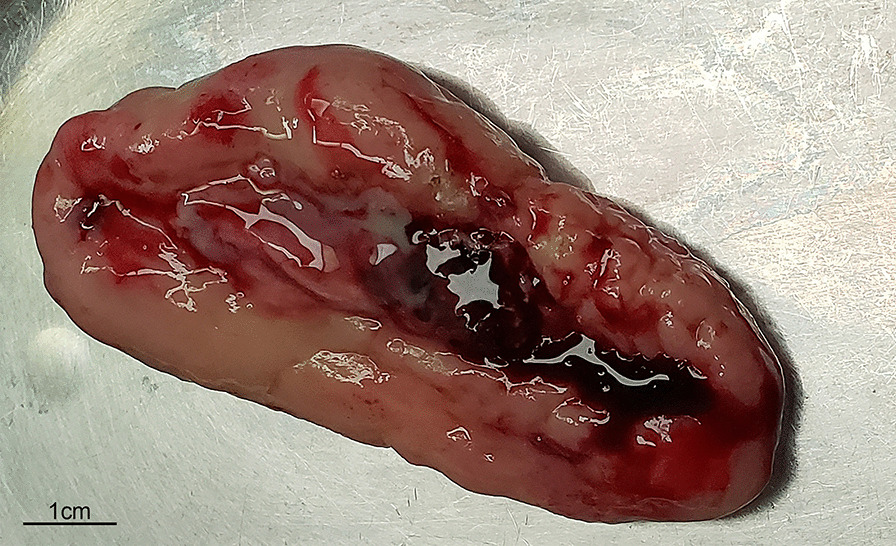
Macroscopic characteristics of the resected tumor, which was located in the middle and lower part of the thoracic esophagus, and measured 8.5*4*2.7 cm

**Fig. 3:**
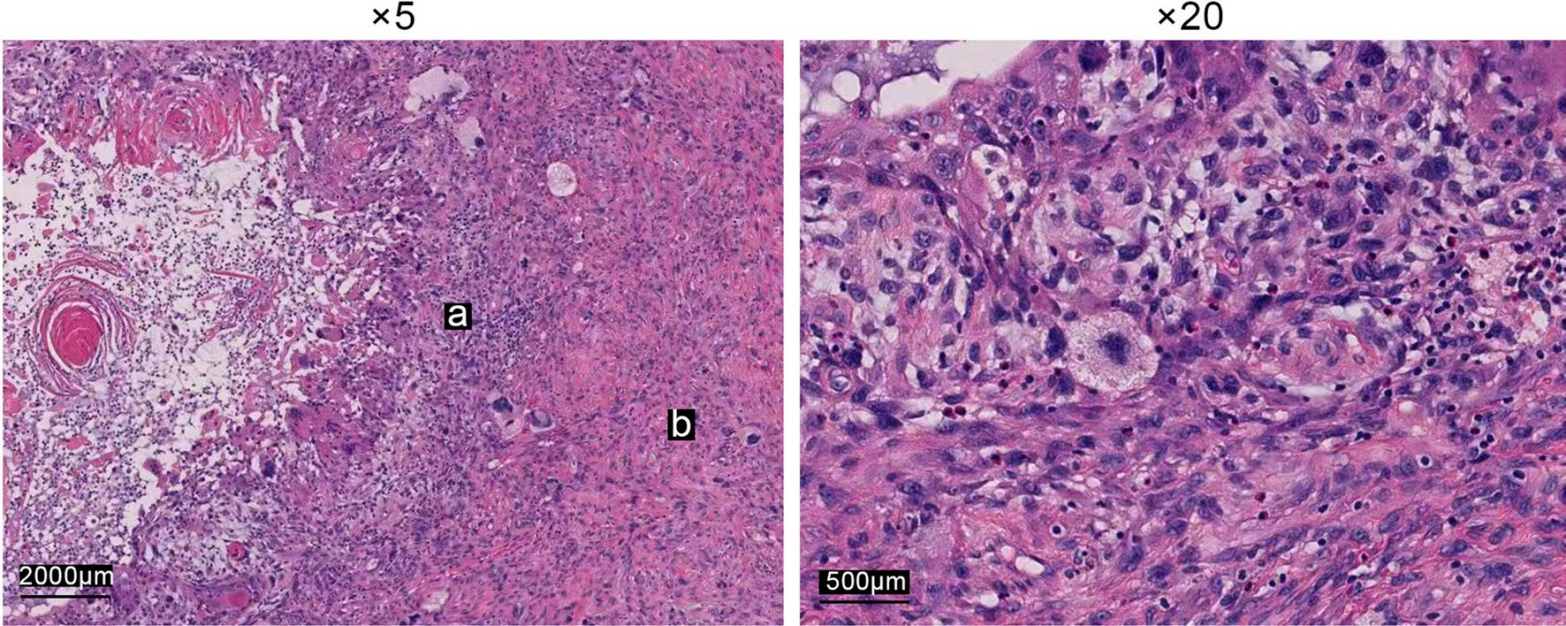
Histopathological findings of the resected tumor. The low-power field image shows that both a carcinomatous component (*a*) and a sarcomatous component (*b*) are found in the tumor. The high-power field image shows that the squamous cell carcinoma component exhibits dense hyperchromatic nuclei with a high nucleocytoplasmic ratio. The sarcomatous component shows spindle-shaped cells with nuclear atypia

**Fig. 4:**
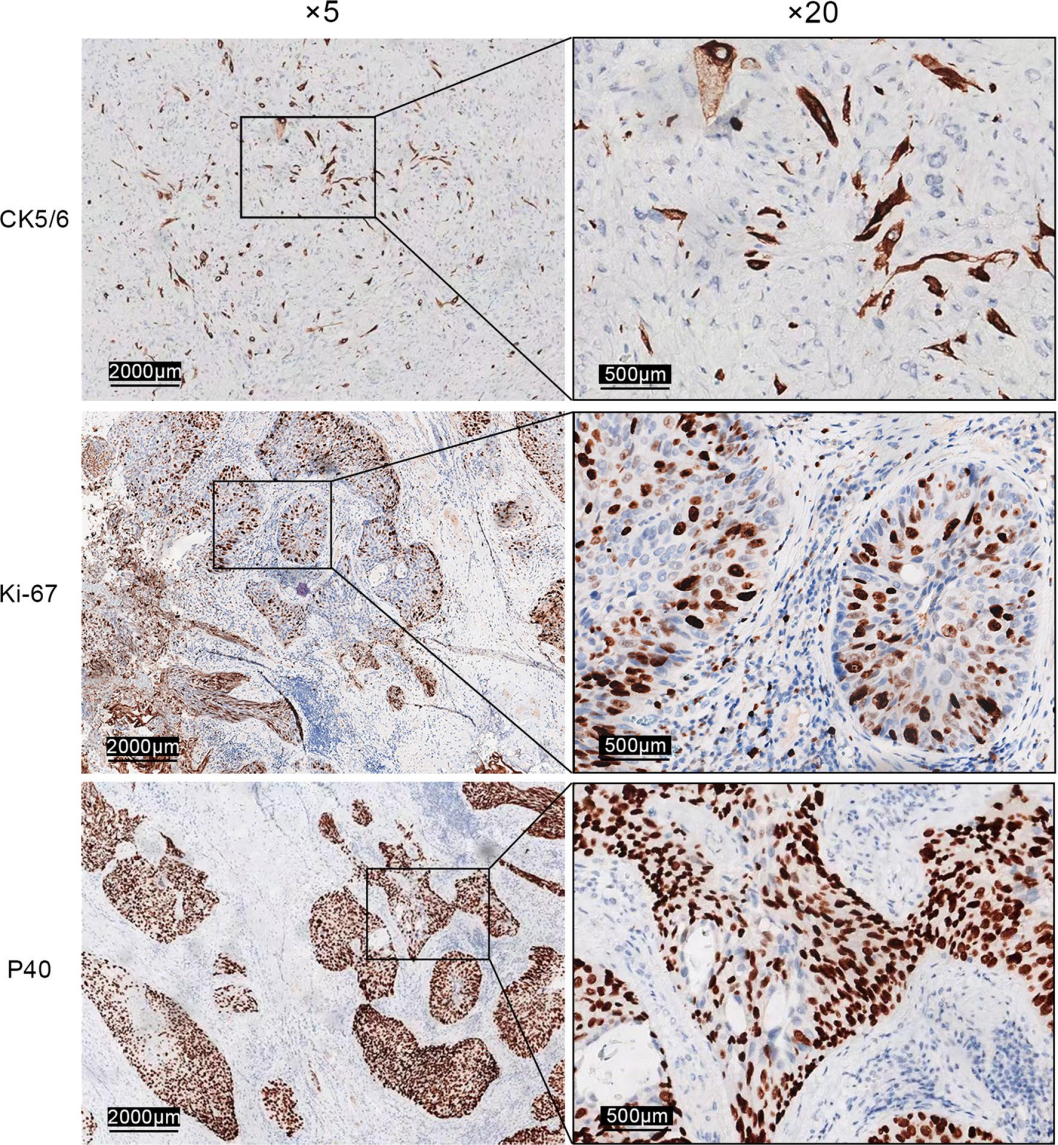
Immunohistochemical features of the resected tumor. Marked immunoreactivity of CK5/6, an epithelial and basaloid marker, was observed in the squamous carcinoma component. Marked immunoreactivity of Ki-67, a biomarker that reflects the proliferative activity of a cell, was observed in approximately 70% of the squamous carcinoma nuclei, which indicates the malignant proliferative growth pattern of the tumor. Marked immunoreactivity of P40 (a tumor suppressor protein) was observed in the carcinomatous component

**Fig. 5:**
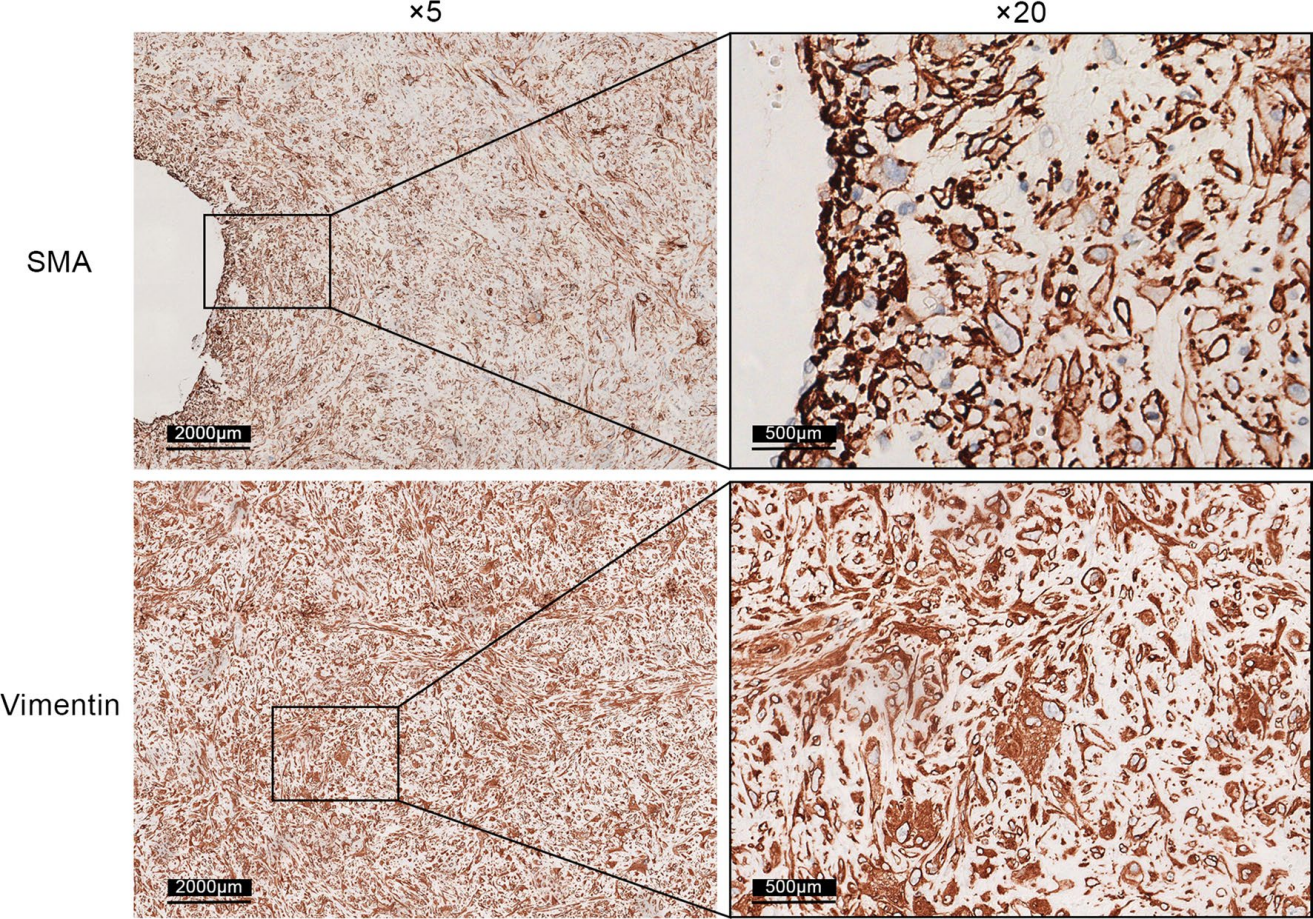
Immunohistochemical features of the resected tumor. Marked SMA (marking smooth muscle cells) immunoactivity in sarcomatous components. Marked immunoreactivity of Vimentin (a mesenchymal marker) was observed in the cytoplasm in the sarcomatous component

The tumor was a large, hard polypoid tumor with a broad pedicle and ulcerations on its smooth surface (Fig. 2), and it almost filled the middle-lower thoracic esophagus. The tumor was 8.5*4*2.7 cm in size and exhibited a cystic structure enclosing grayish-yellow mucus. The resected esophagus was 13 cm in length. Hematoxylin & eosin (H&E) staining (Fig. 3) and immunohistochemical (IHC) analysis (Figs. 4, 5) were performed. H&E staining showed both carcinomatous and sarcomatous elements. In the basal part of the squamous epithelium, atypical cells with hyperchromatic nuclei were found. Since the basal lamina was intact, the lesion was considered a carcinoma in situ. The sarcomatous component was located adjacent to the carcinomatous lesion, and consisted of atypical pleomorphic cells with an irregular arrangement. Both the superior and inferior margins of the resected esophagus were free from malignancy. The pathological report revealed that the tumor invaded the submucosal layer of the esophagus without metastasizing to any of the three groups of dissected regional lymph nodes (upper abdominal lymph nodes, inferior carina lymph nodes and lower periesophageal lymph nodes). The IHC analysis report indicated that the sarcomatous element was Vimentin ( +), SMA ( +), CD68 (+), Ki-67 (+ 20%), S100 (−), CD34 (−), Desmin (−), CD56 (−), Bcl-2 (−), Calponin (−), B-Catenin (−), and ALK (CD246) (−) and that the carcinomatous component was CK5/6 (+), P40 (+), and Ki-67 (+ 70%). IHC examination indicated that the sarcomatous component did not display any signs of differentiation, and the sarcomatous lesion was thus diagnosed as undifferentiated pleomorphic sarcoma based on the IHC and H&E staining. According to the Japanese Classification of Esophageal Cancer, 11th Edition, the patient was finally diagnosed with ECS, clinical stage T1bN0M0, and pathological stage I [6]. For postoperative management, supportive treatments, including preventive anti-infection treatment, hemostasis and inhibition of gastric acid secretion, were employed. Subcutaneous insulin injection was also employed to control the patient’s blood glucose level. Seven days after the operation, a meglumine diatrizoate upper gastrointestinal contrast study was conducted, and no anastomotic leakage was observed. Nine days after the surgery the patient was allowed to consume an oral liquid diet. Eleven days after the surgery, the patient was able to consume a semiliquid diet and was discharged from the hospital with both the pleural and mediastinal drainage tubes removed. The latest follow-up of the patient before the manuscript was submitted was in June 2022. The patient was able to consume common foods without experiencing severe complications, and no signs of recurrence or metastasis were found.

## Discussion

The first case of esophageal carcinosarcoma was reported by Hansemann in 1904 [7]. The incidence of ECS is very low; Wu reported a large cohort study between 1973 and 2011 in which esophageal sarcoma accounted for 0.3% of all malignant esophageal tumors and 32% of the esophageal sarcomas were ECS [8]. ECSs commonly form as large polypoid tumors attached to the wall of the esophagus via a pedicle, with or without ulceration. A study conducted by Chino indicated that the collagenous stroma of the sarcomatous component contributes to the polypoid morphology of the tumor and that the carcinomatous component of the tumor leads to an ulcerative pattern similar to that of ordinary squamous cell carcinomas [2, 9]. Several hypotheses have been proposed regarding the etiology of esophageal carcinosarcoma. Lane suggested that the sarcomatoid component arises from fibroblastic proliferation stimulated by the adjacent carcinomatous component [10]. Jun reported a case in which the sarcoma cells were morphologically different from the carcinoma cells and did not express any epithelial markers, indicating that some ECSs result from the simultaneous but independent occurrence of a carcinoma and a sarcoma [11]. Matsumoto found that an original clone of squamous cell carcinoma could acquire a sarcomatous phenotype through progressive accumulation of genetic alterations and from carcinosarcoma [12].

ECS shares similar clinical manifestations with ESCC. Both malignancies have dysphagia as the most frequent symptom and are sometimes accompanied by chest pain, odynophagia, hematemesis and weight loss [13, 14]. A statistical analysis conducted by Schizas revealed that most ECSs are located in the middle esophagus (56.9%), followed by the lower esophagus (23.5%) and the upper esophagus (19.6%). ECS most frequently develops in middle-aged men with a history of smoking and/or alcohol abuse, as indicated by further investigations. Imaging examinations are crucial in the diagnosis and management of ECS. An upper gastrointestinal X-ray contrast study can reveal the location of the lesion, while CT and positron emission tomography (PET) play a pivotal role in detecting local invasion and lymphatic/distal metastasis to assist TNM staging [1]. In the reported case, the X-ray barium meal test and contrast-enhanced CT scan indicated the primary invasion of the neoplasm and guided the subsequent therapeutic plan. Considering the financial cost of making an appointment for the PET scan, the patient and his relatives refused PET scan. IHC analysis is the gold standard for the diagnosis of carcinosarcoma. There are various specific markers for the carcinomatous elements such as CEA, CD56 and synaptophysin. Regarding the sarcomatous component, desmin, SMA and vimentin are sensitive markers [1]. IHC analysis of the reported case showed no transitional zone with both CK5/6 and vimentin immunoreactivity, and thus, the sarcomatous component was not considered to be derived from the same monoclonal origin as the carcinomatous component. In addition to these traditional diagnostic methods, endoscopic ultrasonography (EUS) and measurement of several serum cytokines have been applied by some scholars to evaluate ECS [15, 16].

The therapeutic management of primary ECS is decided by the location, pathological type, TNM stage and pathological stage of the neoplasm. Endoscopic treatment might be used to manage on ECSs with smaller sizes that are limited to the mucosal/submucosal layer without lymph node or distal metastasis, as well as those in patients with poor health, as these individuals may have a high risk of perioperative complications or contraindications to major surgery [17, 18]. Complete resection of the esophagus with lymphadenectomy of locoregional nodes is still recommended as the best potentially curative treatment. A multicenter European study showed that 21 patients accepted transthoracic esophagectomy over 29 years, with 5-year overall survival (OS) and disease-free survival (DFS) rates of 35% and 33%, respectively, and a total recurrence rate of 48% [5]. Wu conducted a study that included data from 18 state and regional centers in 2015 and indicated that surgery showed a significant OS advantage in ECS [8]. Regarding the surgical approach, Lv investigated the outcomes of ten patients who underwent uniport VATS for esophageal surgery and found that none of the ten patients underwent conversion to open surgery, and no serious complications occurred [19]. Hasan reported an 18-patient investigation in which 15 patients with esophageal cancer who underwent uniport VATS showed no perioperative complications, and three patients experienced leakage. He concluded that intrathoracic esophagogastric anastomosis is a pivotal step to reduce perioperative leakage [20]. Chemotherapy and radiotherapy have also been reported by some scholars for ECS treatment. Xu recommended that chemoradiotherapy associated with endoscopic management could be considered as an alternative to surgery for patients with ECS when surgery is not feasible and Kimura reported a case in which the ECS tumor disappeared after palliative radiotherapy (45 Gy/15 fr) alone [14, 21].

Regarding the prognosis of ECS compared to ESCC, there is still a controversy. Some scholars believe that ECS has a better prognosis than ESCC. Wang analyzed the clinical outcomes of 33 patients and found that the median OS time for all patients with esophageal carcinosarcoma was 43.5 months and that the 1-year, 3-year, and 5-year OS rates were 74%, 57%, and 48%, respectively, better than those of patients with ESCC in their center [3]. Another multicenter analysis conducted by Zhang also revealed that ECS has a better prognosis, with a 5-year OS time of 43 months, longer than that for ESCC, which was 37.5 months [22]. The reasons that ECS may have a better prognosis than ESCC include its low malignant potential and its intraluminal growth pattern, which can lead to early diagnosis and treatment [14]. However, some scholars hold the opposite opinion, i.e., that ECS does not have a better prognosis than ESCC. Lyomasa conducted a twenty-case study in 1990 in which the clinical outcomes of the 20 carcinosarcoma patients were compared with those of 773 patients with squamous cell carcinoma; the results indicated that although the three-year survival rate of ECS is higher than that of squamous cell carcinoma, there is no significant difference in the five-year survival rate between the two neoplasms. Moreover, ECS has a higher frequency of lymph node recurrence and hematogenous metastasis [4]. Sano reported that hematogenous metastasis was more common in ECS than in ESCC. Sano also found that more organ metastases consisted of sarcomatous components [23]. These findings indicate that early diagnosis and surgical treatment with lymphadenectomy are essential to improve the prognosis of ECS.

## Conclusion

In summary, we reported a case of ECS in which the polypoid tumor comprised squamous cell carcinoma in situ and undifferentiated pleomorphic sarcoma. The patient underwent Ivor-Lewis subtotal esophagectomy with intrathoracic anastomosis, without recurrence or metastasis at the 14-month follow-up. Our report suggests that ECS should be considered with regard to the diagnosis of esophageal tumors. Surgical treatment should be conducted in patients diagnosed with ECS, and VATS esophagectomy could be an alternative to open surgery with fewer complications.

## Data Availability

All data generated or analyzed during this study are included in this published article.

## Abbreviations

ECS: Esophageal carcinosarcoma
ESCC: Esophageal squamous cell carcinoma
CT: Computed tomography
VATS: Video-assisted thoracic surgery
H&E: Hematoxylin & eosin
IHC: Immunohistochemical
PET: Positron emission tomography
EUS: Endoscopic ultrasonography
OS: Overall survival
DFS: Disease-free survival
